# No Job Demand Is an Island – Interaction Effects Between Emotional Demands and Other Types of Job Demands

**DOI:** 10.3389/fpsyg.2019.00873

**Published:** 2019-04-18

**Authors:** Martin Geisler, Hanne Berthelsen, Jari J. Hakanen

**Affiliations:** ^1^Centre for Work Life and Evaluation Studies, Malmö University, Malmö, Sweden; ^2^Department of Psychology, University of Gothenburg, Gothenburg, Sweden; ^3^Faculty of Odontology, Malmö University, Malmö, Sweden; ^4^Finnish Institute of Occupational Health, Helsinki, Finland

**Keywords:** emotional demands, job demands, interaction, quality of work, meaning in work, human service

## Abstract

Emotional demands are an inevitable feature of human services, and suggested to be a defining antecedent for workers’ stress and ill health. However, previous research indicate that emotional demands can have a favorably association to certain facets of human service workers’ motivation and well-being. Furthermore, recent research report that the effect of emotional demands on workers’ health and well-being seem to be contingent on the parallel level of other job demands. Still, initial investigations of interaction effects between emotional demands and other types of job demands have primarily focused on negative outcomes in terms of stress-related concerns and absenteeism. The present study investigated interaction effects between emotional demands and other types of job demands in relation to positive outcomes. In a larger sample of human service workers (social workers, *n* = 725), interaction effects were investigated between emotional demands and other job demands (quantitative demands, work pressure, and role conflict) for meaning in work and quality of work. Hypotheses stated that other job demands would moderate the relationship between emotional demands and positive outcomes, so that emotional demands would have a positive relation (i.e., act as a challenge) when the level of other demands is lower, but have a negative relation (i.e., act as a hindrance) when the level of other demands is high. Overall, the results provided support for the idea that emotional demands may act as a challenge. We found small but significant interaction effects between emotional demands and work pressure – in relation to meaning of work, as well as between emotional demands and quantitative demands, work pressure, and role-conflict, respectively – in relation to quality of work. Yet, the results did not support the assumption that emotional demands act as a hindrance when the level of other types of job demands is high. In sum, the results contribute by showing that emotional demands may promote human-service workers’ job attitudes when the level of parallel job demands is lower. We discuss the contribution of the study and the potential practical implications of the results, and give some suggestions for future research.

## Introduction

People confront numerous demands at work. The Job Demands-Resources (JD-R) theory proposes that high levels of job demands may exhaust employees’ physical and psychological energy, ultimately causing various health problems such as stress, exhaustion, and burnout (Schaufeli and Taris, 2014; Bakker and Demerouti, 2017). These relations are illustrated by the current state of affair in many Human Service Organizations, where increasing levels of job demands, growing health-related concerns, and high turnover rates have been reported during the last few decades (e.g., Mor Barak et al., 2001; Tham, 2007; Frost et al., 2017).

However, many human service occupations are inevitably demanding, especially with regard to emotional demands (Hasenfeld, 2010). This often makes it difficult to decrease this specific type of demand (Bakker and Sanz-Vergel, 2013). Based on this consideration, recent research has explored interaction effects among emotional demands and other types of job demands (van Woerkom et al., 2016; Jimmieson et al., 2017). In brief, this research has reported results in favor of the idea that levels of specific job demands (e.g., workload) affect how emotional demands relate to important outcomes such as sickness absenteeism and various stress-related concerns. Building on these initial insights, the present study makes novel contributions to this field of inquiry by including a broad range of job demands (emotional demands, quantitative demands, work pace, and role conflict), and investigating interaction effects between emotional demands and other types of job demands in relation to positive outcomes in terms of meaning in work and quality of work.

### The Job Demands-Resources Theory

A basic proposition of the JD-R theory is that all job characteristics can be categorized as *job demands* or *job resources* (Bakker and Demerouti, 2014, 2017, 2018). Job demands are defined as those job characteristics that require effort and drain energy (e.g., workload, emotional demands, and role conflict), consequently triggering the *health impairment process*. In contrast, job resources are defined as job characteristics that provide possibilities for achievement and support psychological needs (e.g., social support, feedback, and variation), thereby triggering the *motivational process*.

Regarding interaction effects among job characteristics, previous research on the JD-R theory has primarily attended to how job resources may *buffer* against the effect that job demands assert on health impairment, or how job demands can *boost* the effect that job resources have on motivation (Bakker et al., 2005; Schaufeli and Taris, 2014; Bakker and Demerouti, 2017, 2018). Furthermore, it has been reported that job resources are more likely to have a buffering effect on the stressor–strain relationship when there is a match between types of stressors, resources, and strains (de Jonge and Dormann, 2006; de Jonge et al., 2008). However, it has been noted that interaction effects among job demands have been overlooked, as research mostly has investigated the separate effect of certain job demands or the cumulative effect of multiple job demands (van Woerkom et al., 2016; Jimmieson et al., 2017). This is a limitation, as it is unlikely that the (psychological) effects of different job demands act in isolation. For instance, imagine a social worker who is required to handle emotional distress from clients and co-workers (i.e., emotional demands), while simultaneously being overwhelmed by tasks and duties (i.e., quantitative demands), having many duties that need to be attended to as soon as possible (i.e., work pace), or being confronted with conflicting obligations at work (i.e., role conflict).

### Job Demands as Challenges or Hindrances

Research on the JD-R theory has convincingly demonstrated that job demands are unfavorably related to various types of work-related outcomes (for overviews, see Schaufeli and Taris, 2014; Bakker and Demerouti, 2017, 2018). However, the effects seem to be more complex as job demands, in addition to negative impacts, can have positive consequences for important features at work. Based on the results of meta-analytic tests, an extension of the JD-R theory that distinguishes between *challenge* job demands and *hindrance* job demands has been proposed (Cavanaugh et al., 2000; Lepine et al., 2005). The general assumption of the two-dimensional-stressor framework is that, even though job demands may act as challenges or hindrances, both challenges and hindrances are stressors that ultimately have unfavorable effects on workers’ health (Crawford et al., 2010). Yet, whereas hindrance job demands interfere with work-related functioning and obstruct goal-attainment, challenge job demands may have positive effects on motivation and engagement since they hold opportunities for personal gains or accomplishments (Webster et al., 2010). Indeed, challenges and hindrances have been reported to be oppositely related to work-attitudes (Boswell et al., 2004), positive affect, and work-engagement (Tadic et al., 2015), job satisfaction (Webster et al., 2010), performance (Lepine et al., 2005), and psychological resilience to strain (Crane and Searle, 2016).

Hence, empirical evidence suggests a distinction between challenge and hindrance job demands. Nevertheless, a classification of typical challenges or hindrances is still unclear. For example, some research suggests that work pressure (cf. work pace) may act as a challenge in general (Crawford et al., 2010), while other research reports that work pressure seems to act as a hindrance in certain occupations (Bakker and Sanz-Vergel, 2013). Schaufeli and Taris (2014) encouraged future research to focus on challenges and hindrances, in order to improve the understanding of this redefinition and identify typical challenges. Bakker and Demerouti (2017) repeated this call, proclaiming that uncovering the conditions under which job demands act as hindrances or challenges constitutes a significant and unresolved issue for JD-R research. In order to disentangle this issue, researchers need to attend to the fact that people can interpret demands differently (Searle and Auton, 2015), that a specific demand may act as a challenge in one setting but as a hindrance in another (Bakker and Sanz-Vergel, 2013), and, consequently, that a demand may act as both a challenge and a hindrance in the same setting (Webster et al., 2011).

### Emotional Demands in Human Service Professions

In light of this complexity, investigations of interaction effects among job demands offer one promising avenue for improving the understanding of “typical” challenges and hindrances. Arguably, in line with the notion that the specificity of job demands depends on the occupational setting (Bakker and Demerouti, 2017), identification of typical challenges and hindrances seem to be best pursued within specific occupational sectors and/or certain types of professions. Emotional work, to confront situations that are emotionally strenuous and become emotionally concerned at work (van Woerkom et al., 2016; Jimmieson et al., 2017), is an inevitable and defining aspect of human service occupations (e.g., Maslach and Jackson, 1981; Guy et al., 2010). At the same time, to respond to and actively handle emotional needs is often a basic motive for why people choose to work in human service (e.g., to make a difference, “have a calling”). Thus, emotional demands might not have an adverse effect on certain aspects of well-being (Taris and Schreurs, 2009). Supportive of this, emotional demands can act as a challenge in human services (i.e., health care) by interacting with personal resources to predict work engagement (Bakker and Sanz-Vergel, 2013). Moreover, whether job demands act as challenges or hindrances is suggested to depend on how demands are valued: negatively valued demands act as hindrances whereas positively valued demands may act as challenges (Schaufeli and Taris, 2014). However, although emotional demands might be positively valued, human service professionals generally face these demands while simultaneously confronting other types of demands. Thus, investigating if and how emotional demands relate to both negative and positive outcomes at work when levels of other job demands are high or low may contribute to a better understanding of when and why emotional demands act as a challenge or a hindrance.

### Interaction Effects Among Job Demands

Recently, van Woerkom et al. (2016) noted that research on the JD-R theory has failed to recognize that job demands may interact and have accumulating or attenuating effects on different outcomes. To explore this possibility, they proposed, and found support for the idea, that high levels of workload (cf. quantitative demands and work pace) strengthen the positive (i.e., unfavorable) relation between emotional demands and registered sick-absenteeism among mental health care professionals (van Woerkom et al., 2016).

Furthermore, in three different samples of health-care workers (hospital employees, ambulance service employees, and aged care/disability workers), Jimmieson et al. (2017) investigated two- and three-way interaction effects among emotional demands, time demands, and cognitive demands. The results showed that emotional demands had a deleterious effect on workers’ health and well-being (i.e., psychological strain, job burnout, stress-remedial intentions, sleep problems, and stress-related turnover intentions) when levels of other job demands (cognitive demands and time demands) were high. In contrast, when the levels of other job demands were low, the direction of relation between emotional demands and adverse outcomes was negative (i.e., emotional demands had a buffering effect).

The results reported by van Woerkom et al. (2016) and Jimmieson et al. (2017) indicate the relevance of attending to interaction effects among job demands. Most importantly, the results suggest that reducing levels of one job demand can have attenuating (i.e., favorable) effects on other demands and, in turn, for workers’ health and well-being. This suggestion has promising practical implications for human services, where reducing levels of emotional demands can be difficult. Nevertheless, van Woerkom et al. (2016) only investigated the interaction effect between emotional job demands and workload. In addition, van Woerkom et al. (2016) and Jimmieson et al. (2017) investigated interaction effects among job demands in respect to negative outcomes. Yet, investigating interaction effects between job demands in relation to positive outcomes is important and can add valuable insights for the two-dimensional stressor framework (i.e., challenges and hindrances job demands). The present study investigates this by attending to positive outcomes in terms of meaning in work and quality of work.

### Meaning in Work and Quality of Work

Research on meaning in work has increased during the last decades (Martela and Pessi, 2018). Even though the issue of meaning in work is quite complex, some precursors of this positive state have been specified. Reviewing research on meaning in work, Rosso et al. (2010) discussed how values, intrinsic motivation, beliefs, authenticity, and purpose are key-factors for why and how people experience meaning in their work. In brief, values are points of reference for desired states to which actual experiences are compared, whereas intrinsic motivation refers to the correspondence between one’s internal drive for meaning in work and experiences of actual work. Beliefs, in turn, denote the role or function that work has in a person’s life (e.g., being a calling). Furthermore, authenticity pertains to the degree to which people believe that they act in accordance with their personal values and responsibilities, while purpose concerns the extent that people perceive their work to be significant and to matter (Rosso et al., 2010). Interestingly, results from experimental studies suggest that when people experience that they are helping others in their work this has a positive effect on how meaningful people evaluate their work to be (Blake et al., 2018).

Degree of quality of work, in terms of workers’ perceptions of the level of quality in the services provided, has been reported to have a beneficial relationship to workers’ job performance (McHugh and Stimpfel, 2012), job satisfaction, work engagement, and organizational commitment (Geisler et al., 2019), health and turnover (Castle and Engberg, 2005; Astvik and Melin, 2012). The relevance of attending to service quality as a positive outcome in JD-R research has been noted (Schaufeli and Taris, 2014). In addition, quality in services has been proposed to be of specific importance in the context of public service motivation, the altruistic motivation to serve other people and society (see Bakker, 2015).

Employees’ perceptions of meaning in work can be expected to be positively related to perceptions of quality of work. Human service occupations are distinguished by being “moral work” (Hasenfeld, 2010), and human service workers are generally motivated by a sense of meaning in work (e.g., providing care or service to people in need) and guided by an ambition to provide high-quality work (Berthelsen et al., 2010; Smith and Shields, 2013; Sikka et al., 2015). Hence, the association can be expected to be especially evident in the context of human service, as evaluations regarding the extent that the services provided correspond to the intended purpose and has meaning are likely to be associated with evaluations of levels of quality in the services provided. Supportive of this, perceptions of a shared socio-moral climate and an organizational commitment to other people’s well-being have been shown to be related to employees’ evaluation of work meaningfulness and performance (Schnell et al., 2013). Thus, it seems reasonable that meaning without quality (or quality without meaning) is an antilogy in human services.

Still, even though expected to be related, meaning in work and quality of work cover two specific features of work that are important and appropriate to attend to in order to investigate if emotional demands acts as a challenge or a hindrance in human service occupations. We anticipated that, even if emotional demands constitute a core feature of human service work that to some extent may be positively valued, the effect of emotional demands is likely contingent on the levels of other types of demands. More specifically, emotional demands may be an antecedent for meaning in work and quality of work – when employees have the opportunity to respond to and manage these demands adequately. Recent research indicates that although the levels of job demands are typically high in human service occupations, the employees report high levels of engagement, which suggests that all demands may not be that deteriorating (Hakanen et al., 2018).

Given this, and in line with the suggestion that the nature of job demands depends on how demands are valued (Schaufeli and Taris, 2014), we considered perception of meaning in work and quality of work as two important aspects to attend to in order to understand when and why emotional demands act as a challenge or a hindrance among human service workers.

### The Present Study

The aim of the present study was to make a contribution to JD-R theory (Schaufeli and Taris, 2014; Bakker and Demerouti, 2017) and the emerging exploration of interactions among job demands (van Woerkom et al., 2016; Jimmieson et al., 2017) by investigating two-way interactions between emotional demands and: quantitative demands, work pace, and role conflict in relation to positive outcomes at work (meaning in work and quality of work).

As the literature review shows, emotional demands are an inevitable feature in human service occupations. Furthermore, emotional demands may be positively valued among human service employees, but at the same time be associated with lower levels of well-being and higher levels of ill health. In order to disentangle this contradiction, the present study focused on emotional demands, treated as the independent variable. In addition, as the purpose of the present study was to investigate how levels of specific types of job demands may affect the direction of relations between emotional demands and positive outcomes, interactions were analyzed where quantitative demands, work pace, and role conflict were the respective moderating variables.

#### Hypotheses

*Hypothesis 1*: Quantitative demands moderate the relationship between emotional demands and meaning in work, so that the relation between emotional demands and meaning in work is positive at low levels of quantitative demands, but negative at high levels of quantitative demands.
*Hypothesis 2*: Work pace moderates the relationship between emotional demands and meaning in work, so that the relation between emotional demands and meaning in work is positive at low levels of work pace, but negative at high levels of work pace.
*Hypothesis 3*: Role conflict moderates the relationship between emotional demands and meaning in work, so that the relation between emotional demands and meaning in work is positive at low levels of role conflict, but negative at high levels of role conflict.
*Hypothesis 4*: Quantitative demands moderate the relationship between emotional demands and quality of work, so that the relation between emotional demands and quality of work is positive at low levels of quantitative demands, but negative at high levels of quantitative demands.
*Hypothesis 5*: Work pace moderates the relationship between emotional demands and quality of work, so that the relation between emotional demands and quality of work is positive at low levels of work pace, but negative at high levels of work pace.
*Hypothesis 6*: Role conflict moderates the relationship between emotional demands and quality of work, so that the relation between emotional demands and quality of work is positive at low levels of role conflict, but negative at high levels of role conflict.

## Materials and Methods

### Participants and Procedure

The data for the present study were collected within the municipal social services in one of the larger Swedish cities, as part of a workplace survey. The survey was preceded by a dialogue between the researchers, representatives of the municipality (the human resources department), and the employees (labor unions). The web survey was distributed by email to all 1,044 social workers employed within the municipality and took approximately 20 min to complete. In all, 831 social workers answered the survey (80% participation rate). As the present study focuses on social workers who have direct contact with clients on a daily basis, participants who reported holding a managerial position (*n* = 96), or had not answered this question (*n* = 10), were excluded from the analyses. Hence, the final sample consisted of 725 social workers. The sample was representative of Swedish social workers in terms of *gender* (85% women, compared to 86% in the Swedish social worker population), but indicated a lower average *age* (<25 years = 5%; 25–34 years = 45%; 35–44 years = 26%; 45–54 years = 14%; 55–60 years = 6%; >61 years = 4%, compared to an approximate mean age of 40 years among Swedish social workers: Statistics Sweden, 2018). Furthermore, the sample was differentiated in terms of *professional tenure*: <1 year = 20%; 1–2 years = 20%; 2–5 years = 23%; 5–7 years = 8%; >7 years = 29%). The study was approved by the Regional Ethical Review Board, Lund secretariat (dnr: 2015-476). Informed consent was obtained from all participants. Participants received written information about the research-project in the email and provided with a link to the survey. When the link was activated, the information was given once more and participants were asked to provide their informed consent by actively replying to the mandatory question: “I have been informed about the study and give my informed consent.”

### Materials and Measures

The data were collected by use of specific scales of the validated Swedish medium-length version of the Copenhagen Psychosocial Questionnaire (COPSOQ II: Pejtersen et al., 2010; Berthelsen et al., 2014, 2018). COPSOQ II includes a number of scales pertaining to employees’ perceptions and experiences of their work conditions and health. Items on the COPSOQ were rated on five-point Likert-type scales. In line with current praxis and principle for scoring on the COPSOQ, scores were converted to the scale 0–100 (i.e., 0, 25, 50, 75, and 100), and subscale scores calculated as the mean item score (Kristensen et al., 2005; Pejtersen et al., 2010). If respondents had answered less than half of the questions, the subscale score was set as missing (cf. Pejtersen et al., 2010; Berthelsen et al., 2017b). In addition, the data collection included a scale for assessments of quality of work (Berthelsen et al., 2017a).

#### Independent Variable

*Emotional demands* (ED, Cronbach’s alpha = 0.79). Emotional demands were measured by four items, rated on five-point scales from 1 (“to a very low degree”) to 5 (“to a very high degree”). An item example is: “*Is your work emotionally demanding?*”

#### Moderating Variables

*Quantitative demands* (QD, Cronbach’s alpha = 0.88), *Work pace* (WP, Cronbach’s alpha = 0.88), and *Role conflict* (RC, Cronbach’s alpha = 0.68) was assessed by three items each, and rated on five-point scales from 1 (“*to a very low degree*”) to 5 (“*to a very high degree*”). Item examples are: *“Do you get behind with your work?*” (QD); “*Do you need to keep a high work pace throughout the day?*” (WP); “*Are contradictory demands placed upon you at work?*” (RC).

#### Dependent Variables

*Meaning in work* (MW, Cronbach’s alpha = 0.79) was assessed by three items, and *Quality of work* (QW, Cronbach’s alpha = 0.77) by two items, rated on five-point scales from 1 (“*to a very low degree*) to 5 (“*to a very high degree*”). Item examples are: *“Is your work meaningful?”* (MW); “*To what extent do you find it possible to perform your work tasks at a satisfactory level of quality?”* and “*Are you satisfied with the level of quality in the services that are conducted at your workplace*” (QW).

### Data Analyses

To test the hypotheses, interaction (moderation) effects were analyzed by hierarchical multiple regression using SPSS statistics (version 24). The independent and moderating variables were mean centered and used to compute the interaction terms, in line with recommendations for reducing multicollinearity (Aiken and West, 1991; Frazier et al., 2004). Each regression model included the mean centered variable for emotional demands (i.e., the independent variable), and the mean centered variable of the respective demand (i.e., the moderating variable), as well as the interaction-term calculated by use of the corresponding mean centered variables. Simple slopes were calculated and analyzed based on the sample values (i.e., estimates of population values: *M* – 1 *SD*, and *M* + 1 *SD*). Of note, we also controlled for the potential effect of gender and professional tenure by inserting these variables in the very first step of the multiple regression analyses. However, the contribution of this step was non-significant and therefore we did not include this step in the final analyses.

## Results

### Descriptive Statistics and Correlations

Table 1 reports the descriptive statistics and the correlations for all variables. Emotional demands were found to correlate low to moderately with the other job demands, whereas the correlations between the other job demands were moderate to high. The correlations between other job demands and the dependent variables were negative, whereas we found a positive, weak correlation between emotional demands and quality of work and between emotional demands and meaning in work.

**Table 1:** Descriptive statistics and correlations.

	*M*	*SD*	*Skew*	*Kurtosis*	*N*	1	2	3	4	5	6
1. Emotional demands	71.2	16.1	-0.324	-0.264	725	-					
2. Quantitative demands	50.5	22.0	0.055	-0.511	724	0.31***	-				
3. Work pace	64.7	20.7	-0.206	-0.340	724	0.29***	0.68***	-			
4. Role conflict	45.7	17.1	-0.067	0.195	725	0.20***	0.44***	0.41***	-		
5. Meaning in work	79.1	15.5	-0.639	0.366	724	0.12**	-0.19***	-0.08*	-0.35***	-	
6. Quality of work	62.0	18.3	-0.262	0.364	720	-0.08*	-0.48***	-0.33***	-0.49***	0.49***	-


*^∗^*p* < 0.05; ^∗∗^*p* < 0.01; ^∗∗∗^*p* < 0.001*.

### Hierarchical Multiple Regression Analyses

#### Meaning in Work

**FIGURE 1:**
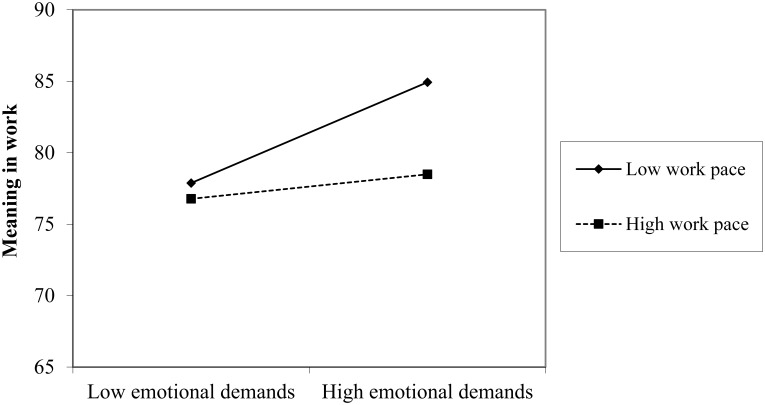
Plot of the two-way interaction effect of emotional demands and work pace on meaning in work.

Table 2 reports the results of the regression analyses for meaning in work and quality of work. With regard to main effects of job demands, the results showed that emotional demands were related to higher reports of meaning in work, whereas quantitative demands, work pace, and role conflict were related to lower reports of meaning in work. No significant interaction effects were found between emotional demands and quantitative demands, or between emotional demands and role conflict. Thus no support was provided for hypothesis 1 or hypothesis 3. However, the interaction effect between emotional demands and work pace was significant. Simple slope test revealed that the positive association between emotional demands and meaning in work was significant at simple slopes of low levels of work pace, *B* = 0.23, *t*(720) = 4.71, *p* < 0.001, but not significant at high levels of work pace, *B* = 0.05, *t*(720) = 4.71, *p* < 0.351 (Figure 1). Hence, the results provided partial support for hypothesis 2, in that emotional demands act as a challenge and have a positive effect on meaning in work at low levels of work pace. No support was provided for the expectation that emotional demands act as a hindrance and have a negative effect on meaning in work at high levels of work pace.

**Table 2:** Hierarchical multiple regression analyses for meaning in work and quality of work.

	Meaning in work				Quality of work			
	*β*	Adj *R*^2^	*ΔR*^2^	*F* change	*β*	Adj *R*^2^	*ΔR*^2^	*F* change
**Model 1**								
*Step 1*		0.070	0.072	28.117***		0.232	0.235	109.705***
Emotional demands (ED)	0.188***				0.062			
Quantitative demands (QD)	-0.250***				-0.496***			
*Step 2*		0.070	0.001	1.075		0.236	0.005	4.572*
ED × QD	-0.038				-0.072*			
**Model 2**								
*Step 1*		0.025	0.028	10.401***		0.107	0.110	44.206***
Emotional demands (ED)	0.141***				0.009			
Work pace (WP)	-0.121**				-0.335			
*Step 2*		0.032	0.008	6.298*		0.112	0.006	4.900*
ED × WP	-0.093*				-0.079*			
**Model 3**								
*Step 1*		0.157	0.159	68.294***		0.235	0.237	111.340***
Emotional demands (ED)	0.201***				0.008			
Role conflict (RC)	-0.392***				-0.482***			
*Step 2*		0.156	0.000	0.398		0.240	0.006	5.696*
ED × RC	0.022				-0.079*			


*^∗^*p* < 0.05; ^∗∗^*p* < 0.01; ^∗∗∗^*p* < 0.001.*

#### Quality of Work

**FIGURE 2:**
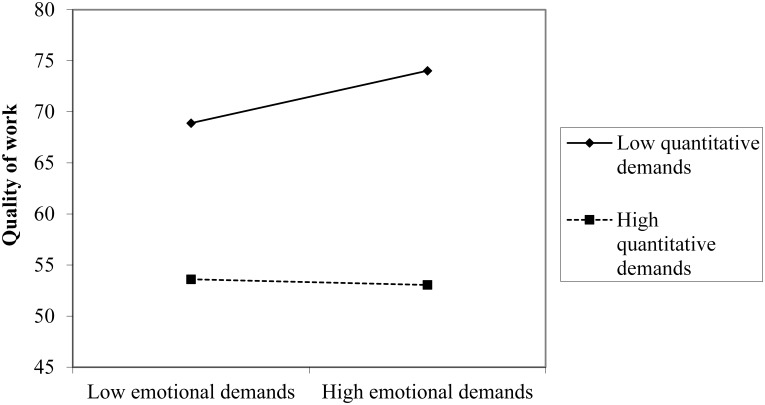
Plot of the two-way interaction effect of emotional demands and quantitative demands on quality of work.

The results for the main effects of job demands showed that emotional demands were not significantly related to quality of work, whereas quantitative demands, work pace, and role conflict were related to lower reports of quality of work (Table 2). Furthermore, the result showed significant interaction effects between emotional demands and quantitative demands, between emotional demands and work pace, and between emotional demands and role conflict. Simple slope analyses for the interaction effect between emotional demands and quantitative demands showed that, at low levels of quantitative demands, emotional demands were positively related to quality of work, *B* = 0.151, *t*(715) = 3.134, *p* = 0.002 (Figure 2). But at simple slopes for high levels of quantitative demands, the association between emotional demands and quality of work was not significant, *B* = -0.01, *t*(715) = -0.147, *p* = 0.883. Thus, the results of the simple slope analyses provided partial support for hypothesis 4, in that emotional demands act as a challenge and have a positive effect on quality of work at low levels of quantitative demands. However, the hypothesized expectation that emotional demands act as a hindrance and have a negative effect on quality of work at high levels of quantitative demands was not supported.

**FIGURE 3:**
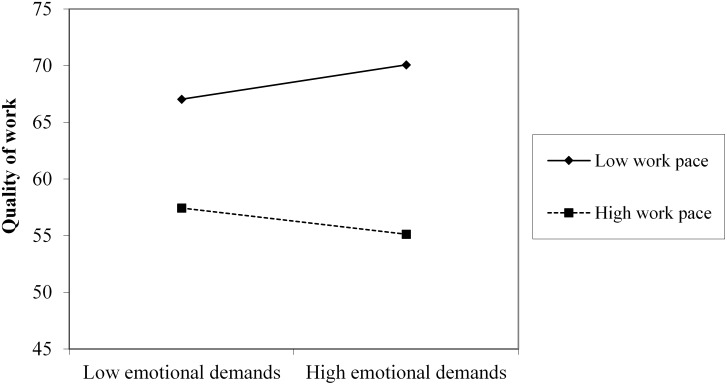
Plot of the two-way interaction effect of emotional demands and work pace on quality of work.

Simple slope analyses for the interaction effect between emotional demands and work pace revealed that emotional demands were positively, but non-significantly, associated with quality of work at low levels of work pace, *B* = 0.097, *t*(715) = 1.815, *p* = 0.070 (Figure 3). Moreover, at simple slopes of high levels of work pace, the relation between emotional demands and quality of work was negative but insignificant, *B* = -0.076, *t*(715) = -1.236, *p* = 0.217. Accordingly, the results of the simple slope analyses did not provide support for hypothesis 5.

**FIGURE 4:**
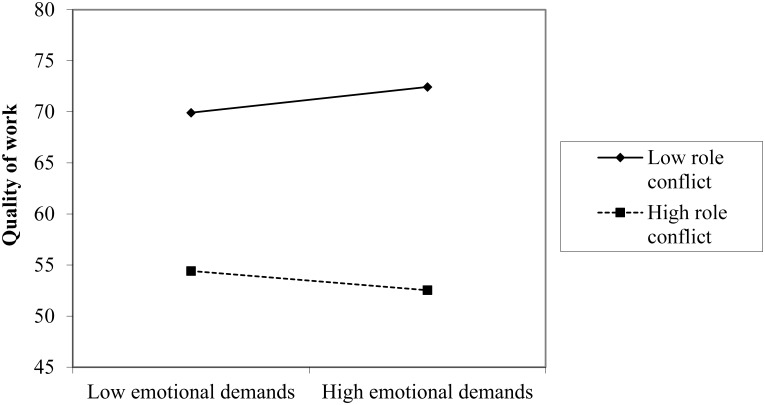
Plots of the two-way interaction effect of emotional demands and role conflict on quality of work.

Simple slope analyses showed that emotional demands had a significant and positive association with quality of work at low levels of role conflict, *B* = 0.099, *t*(716) = 2.028, *p* = 043, whereas at high levels of role conflict, the direction of the association was negative but non-significant, *B* = -0.080, *t*(716) = -1.377, *p* = 0.169 (Figure 4). The results provided partial support for hypothesis 6, in that emotional demands act as a challenge and have a positive effect on quality of work at low levels of role conflict. Yet, the expectation that emotional demands act as a hindrance and have a negative effect on quality of work at high levels of role conflict was not supported.

## Discussion

The purpose of the present study was to investigate interaction effects between emotional demands and other types of job demands in relation to positive outcomes. The aim of this investigation was to contribute to the JD-R theory (Schaufeli and Taris, 2014; Bakker and Demerouti, 2017, 2018), by enhancing the understanding of interaction effects among job demands. In a sample of human service professionals, we investigated whether the effect of emotional demands on workers’ evaluation of meaning in work and quality of work is contingent on the level of other types of job demands. Specifically, we hypothesized that emotional demands would be positively related to meaning in work and quality of work (i.e., act as a challenge), when the level of other types of job demand was low – but that emotional demands would be negatively related to meaning in work and quality of work (i.e., act as a hindrance), when the level of other types of job demand was high.

Overall, the results provided partial support for four out of the six stated hypotheses. Significant interaction effects were found for the relationship between emotional demands and meaning in work with respect to work pace (*hypothesis 2*), whereas significant interaction effects were found for the relationship between emotional demands and quality of work with regard to quantitative demands (*hypothesis 4*), work pace (*hypothesis 5*), and role conflict (*hypothesis 6*). However, while significant interaction effects were found, our expectation of full moderation was not met. Emotional demands had a positive effect on meaningfulness and perceived quality of work when the level of other job demands was low, but the expected corresponding negative effect was not found in relation to high levels of other job demands.

Our findings demonstrate that it is relevant to investigate interaction effects among job demands in relation to positive outcomes. Specifically, our results show that, among human service workers, emotional demands may act as a challenge and be associated with higher evaluations of quality of work and meaning in work, particularly when levels of other job demands are lower. This is in line with our expectations and with the notion that emotional demands can have a beneficial relation to job attitudes and specific aspects of well-being (Taris and Schreurs, 2009). In addition, the findings corroborate the importance of attending to emotional demands in order to understand human service work (e.g., Guy et al., 2010; Bakker and Sanz-Vergel, 2013).

Furthermore, our results specifically showed that other types of job demands moderate the relationship between emotional demands and quality of work. As emotional demands are an inevitable feature (Guy et al., 2010) and quality of work is an important concern in human service (Astvik and Melin, 2012; McHugh and Stimpfel, 2012; Bakker, 2015; Geisler et al., 2019), this result is intriguing. The present findings contribute to the notion that, as the level of emotional demands is often difficult to adjust (Bakker and Sanz-Vergel, 2013), efforts and interventions aimed at reducing the level of other types of job demands may be expected to have positive spill-over effects by providing opportunities for more favorable effects of emotional demands not only in regard to negative outcomes (van Woerkom et al., 2016; Jimmieson et al., 2017) but also to positive outcomes.

Still, our results indicate that emotional demands seem to provide a sense of meaning in work for human service workers in general, possibly by offering an opportunity to serve people in need (e.g., Smith and Shields, 2013; Bakker, 2015). This also fits well with the idea that whether a job demand acts as a challenge or a hindrance depends on how it is valued in the specific context/occupational group (Schaufeli and Taris, 2014). That is, emotional demands are a characteristic of human service jobs that are positively valued and contribute to the purpose and meaning in work, but may only have this function for quality of work when the levels of other job demands do not interfere.

Moreover, our results did not provide support for the hypothesized expectations that emotional demands would act as a hindrance, and have a negative relationship to positive outcomes, when levels of other types of job demands are high. The very nature of human service jobs may explain the unexpected findings. As our literature overview makes evident, emotional demands are a distinctive feature of human service jobs and is often a basic motive for why people choose to work in human service. Thus, human service professionals anticipate being confronted with emotional demands and to likely regard them as rather positive. Therefore, and as suggested by our results, emotional demands *can* act as a challenge but *may not* act as a hindrance in relation to *positive* outcomes.

In addition, our results contribute by adding insights to the notion that working life is highly complex. Not only do people have to handle the interests of different stakeholders, or obtain, maintain, and cultivate necessary resources, but they also need to handle numerous, parallel, and sometimes conflicting demands. The JD-R theory provides a heuristic and flexible framework for assessment and improved understanding of processes associated with both motivational and health-related outcomes (Schaufeli and Taris, 2014). However, conceivable intricate effects among different job demands have generally been overlooked in previous JD-R research. The present results inform the emerging literature on interaction effects among job demands (van Woerkom et al., 2016; Jimmieson et al., 2017), and the calls for investigations of “typical” challenges and hindrances (Schaufeli and Taris, 2014; Bakker and Demerouti, 2017). For instance, previous studies have reported that when emotional demands are combined with high levels of workload (van Woerkom et al., 2016), cognitive demands, or time demands (Jimmieson et al., 2017), the interaction effects relate to indicators of workers’ health. However, in this previous research, the importance of interaction effects has mainly been explored in relation to negative outcomes in terms of indicators of stress and exhaustion, such as sickness-absenteeism (van Woerkom et al., 2016), psychological strain, and stress-remedial intentions (Jimmieson et al., 2017). That is, interaction effects in relation to positive outcomes have been largely overlooked. Yet, Jimmieson et al. (2017) did investigate interaction effects among job demands in respect to job satisfaction, and found a significant three-way interaction effect in one of their three studies (Study 3). Thus, Jimmieson et al. (2017) reported that emotional demands only had a negative effect on job satisfaction when levels of both cognitive and time demands were high, but seemed to have a buffering effect on job satisfaction when the levels of at least one of the other type of demands was low. The findings of the present study demonstrate that two-way interaction effects among emotional demands and other types of demands can be expected in relation to positive outcomes. Our results suggest that emotional demands can act as a challenge with regard to important positive outcomes, but that the effect is contingent on the levels of other type of job demands – corroborating the proposition of the two-dimensional stressor framework (Crawford et al., 2010), and contributing novel insights to the investigations of “typical” challenges (Schaufeli and Taris, 2014; Bakker and Demerouti, 2017).

In sum, our results suggest that when human service workers meet emotional demands, and are able to attend to these demands (e.g., at lower work pace), they can have a positive effect on evaluations of the quality of the work performed and, to some extent, the meaning in work. Arguably, one possible explanation for these results is that emotional demands are a job characteristic that is positively valued, supporting intrinsic motivation, and affirming human service workers’ personal beliefs about the core purpose of their work (Hasenfeld, 2010; Rosso et al., 2010).

### Practical Implications

The results inform managers and human-resource practitioners by showing that emotional demands can have a positive effect on human service workers’ perceptions of meaning in work and quality of work, at lower levels of other types of job demands.

Although the results of the present study need to be replicated by subsequent research, and should be interpreted with caution due to the small effects observed, the results show promise for future practical implications. For instance, the results indicate that if human service workers are provided with working conditions that allow them the opportunity to attend to and manage emotional demands, that is, by keeping other types of job demands at a reasonable level, it may increase their positivity toward work. The significance of this is underlined by the fact that emotional demands are an inevitable feature of human service occupations (Guy et al., 2010; Hasenfeld, 2010).

### Limitations and Future Research

The present study has a number of limitations. First, the data are cross-sectional and based on self-reports. Thus, the results should be interpreted with potential common method biases in mind (Podsakoff et al., 2003; however, cf., Spector, 2006). Secondly, although significant interaction effects were found among emotional demands and other types of job demands, the effect of the observed interactions was quite small. This is a general issue and in line with previous research on interaction effects (for a discussion, see Taris, 2017; however, cf. Hakanen et al., 2017). Related to this, emotional demands were only found to have a low to moderate relationship with the dependent variables. Moderator effects have more power when the relation between the predictor and the dependent variable is strong, but moderators are usually examined when the relationship between a predictor and an outcome is weak, which in turn explain why interaction effects tend to be small (Frazier et al., 2004). Still, simple slope analyses reveal the effect of the interaction at different levels of the moderator. In light of these results, the effects of the observed interactions are not arbitrary (e.g., Bakker et al., 2010), but contribute to the basic understanding of how emotional demands relate to human service workers work attitudes depending on the parallel level of other types of job demands.

The present study used *a priori* categorizations of job demands. The appropriateness of this approach has been questioned by previous research on the two-dimensional stressor framework (i.e., challenges and hindrances: Searle and Auton, 2015). If possible, future research could assess how workers appraise (the impact of) different characteristics of the job in order to investigate “typical” challenges and/or hindrances in terms of positively/negatively valued demands/resources (Schaufeli and Taris, 2014). Furthermore, meaning in work and quality of work were assessed by brief, but validated, measures, composed by three and two items, respectively. Yet, as both meaning in work and quality of work are multidimensional constructs, further research using more comprehensive (and/or multifaceted) measures is needed. In addition, the present study is based on self-reports and analyses of statistical significance. It could be discussed whether this approach is able to fully capture the complex psychological processes elicited when workers confront multiple job demands in real life. If possible, an interesting avenue for future research would be to explore “interaction effects” using a qualitative approach. The present sample consisted of a high proportion of women (85%). This proportion is in line with the proportion in the population (i.e., 86% women: Statistics Sweden, 2018). Previous research has reported support for interaction effects between emotional demands and other types of job demands in more gender-balanced samples (e.g., van Woerkom et al., 2016; Jimmieson et al., 2017). However, future research should try to replicate the results of our study in gender-balanced sample and other professional groups. In addition, even though human service workers operating in different welfare systems seem to have a shared view of the core aspects of their job (e.g., Frost et al., 2017), future research should investigate whether the results of the present study can be replicated in other samples and cultural settings.

Finally, in line with the current direction within JD-R research, future studies could investigate the extent to which interaction effects among job demand differ due to individual differences (i.e., personal resources), or organizational factors (e.g., team or organizational level: Bakker and Demerouti, 2018). In this regard, it can be noted that recent research reports that the effect of emotional demands depend on the interplay between personal and team resources (Loi et al., 2016), that training of emotion regulation skills (i.e., a personal resource) may help workers to deal with emotions and enhance well-being (Buruck et al., 2016), and that personal resources can buffer against the negative effects of job demands (e.g., emotional dissonance) on workers’ well-being (Hakanen et al., 2017).

## Conclusion

All in all, this study suggests that emotional demands can contribute to positive outcomes in human service occupations. Previous research has mainly considered emotional demands as a core demand in human service (e.g., Hasenfeld, 2010), but the present study supports the idea that the effect of emotional demands may be contingent on the level of other types of demands.

## Ethics Statement

The study was approved by the Regional Ethical Review Board, Lund Secretariat (dnr: 2015-476), and informed consent was obtained from all participants.

## Author Contributions

All authors listed have made a direct and intellectual contribution to the article and approved the final version for publication. MG developed the hypotheses, analyzed the data, and wrote the first draft. HB conducted the data collection and contributed to the development of the hypotheses. JH contributed to the development of the hypotheses as well as the interpretations and the discussion of the findings.

## Conflict of Interest Statement

The authors declare that the research was conducted in the absence of any commercial or financial relationships that could be construed as a potential conflict of interest.
